# Functional insights into aberrant brain responses and integration in patients with lifelong premature ejaculation

**DOI:** 10.1038/s41598-017-00421-3

**Published:** 2017-03-28

**Authors:** Bing Zhang, Jiaming Lu, Jiadong Xia, Fangfang Wang, Weiping Li, Fei Chen, Youfeng Han, Yun Chen, Bin Zhu, Zhao Qing, Xin Zhang, Yutian Dai

**Affiliations:** 1 Department of Radiology, The Affiliated Drum Tower Hospital of Nanjing University Medical School, Nanjing, Jiangsu China; 2 Department of Andrology, The Affiliated Drum Tower Hospital of Nanjing University Medical School, Nanjing, Jiangsu China; 3 Department of Urology, The First Affiliated Hospital of Nanjing Medical University, Nanjing, Jiangsu China; 4 Department of Radiology, The Affiliated Yancheng Hospital of Southeast University Medical College, Yancheng, Jiangsu China

## Abstract

Even though lifelong premature ejaculation (PE) is highly prevalent, few studies have investigated the neural mechanisms underlying PE. The extent and pattern of brain activation can be determined through a version of functional magnetic resonance imaging (fMRI) with erotic picture stimuli (task fMRI) and a resting-state fMRI (rs fMRI). We showed that the brain activity in the left inferior frontal gyrus and left insula was decreased both during the task and in the resting state, while there was higher activation in the right middle temporal gyrus during the task. Higher functional connectivity was found in PE between those three brain areas and the bilateral middle cingulate cortex, right middle frontal gyrus and supplementary motor area. Moreover, the brain activity had positive correlation with clinical rating scales, such as intravaginal ejaculatory latency time (IELT) and the Chinese Index of Premature Ejaculation (CIPE). These findings revealed that brain responses and functional integration in certain brain areas are impaired in cases of PE, which was consistently supported by multiple measurements obtained using a task and rs fMRI approach.

## Introduction

Premature ejaculation (PE) is one of the most common male sexual dysfunctions, affecting approximately 20–38% of men worldwide^1^. Using an evidence-based unified definition, the International Society for Sexual Medicine defines male sexual dysfunction as ejaculation that always or nearly always occurs prior to or within 1 min of vaginal penetration (lifelong PE) or a clinically significant and bothersome reduction in latency time, often to approximately 3 min or less (acquired PE)^2^.

Previous studies have shown that the etiology of PE is complicated and involves a complex interaction of both psychological and biological factors^3^. Anxiety regarding sexual function is a common precipitating factor. Other factors, such as a history of sexual abuse, attitudes towards sex among peers and certain personal factors, including a poor body image, depression or relationship problems, could have a negative impact on sexual performance^4^. Additionally, there are a number of biological factors that may contribute to PE, including abnormal testosterone or prolactin levels^5^, disturbances in central serotonergic neurotransmission^6^, abnormal reflex activity in the ejaculatory system, and penile hypersensitivity, among other factors. However, the underlying neuronal mechanism of PE is still unclear.

Several studies using functional magnetic resonance imaging (fMRI) have examined brain activation during sexual stimulation in healthy people. During exposure to sexual stimuli, increased signals in the parietal lobes, temporal lobes, parieto-occipital sulcus, superior occipital gyrus, anterior cingulate gyrus, insula, amygdala, and septal areas^7–9^ have been observed; the activation of these areas was highly correlated with the levels of perceived sexual arousal, which were related to the perceived urge to perform sexual actions^8, 10^. In addition, in healthy subjects, decreased signals were recorded in the right posterior cingulate gyrus and the left precuneus^7^ with exposure to the visual stimuli of sexual photographs, and the deactivated brain areas may be related to focusing attention on the sexually arousing stimuli^7, 11^. In lifelong PE patients, a study using electroencephalography showed that neuronal activity in the right para-hippocampal gyrus and the left middle temporal gyrus decreased with exposure to visual erotic stimuli^12^. One probable physiological mechanism is the abnormal activity of certain brain areas^13, 14^ in PE patients, although the brain structures may be intact^15, 16^. Therefore, it is intriguingly to systemically investigate possible abnormal brain activity in PE patients among the whole brain. However, such study is still scarce.

This study attempted to address this important gap in the literature by investigating brain responses, spontaneous reactions and the integration of functional brain connectivity in patients with lifelong PE. Brain responses during a task were calculated with the precision of the parameters (beta) estimated from a general linear model (GLM)^17^. Spontaneous activity was measured via regional homogeneity (ReHo) using resting-state fMRI (rs fMRI)^18^, which reflects spontaneous neuronal activity and the physiological processes of the human brain^19^. ReHo reflects the temporal homogeneity of the regional blood oxygen level-dependent (BOLD) signal. Thus, an altered ReHo may be related to temporal changes in the spontaneous neural activity of a certain region. Furthermore, to evaluate integration within the entire brain, functional connectivity (FC) was introduced to describe which brain areas correlated with the brain responses.

Here, we combined task and rs fMRI analyses to investigate the evolution of brain patterns in lifelong PE patients. Our hypotheses were as follows: 1) brain responses to visual erotic picture stimulation in PE patients are altered compared to those in normal control (NC) subjects; 2) the whole-brain ReHo and FC in a resting-state are reorganized in PE patients; and 3) the brain activation and spontaneous activity changes may be related to the disease severity.

## Results

### Demographic and Clinical Data

The demographic, psychiatric, and behavioral features for lifelong PE patients and NC subjects are listed in Table 1. As shown, the groups did not differ in age (NC: 27.87 ± 3.78 years, PE: 27.95 ± 4.52 years, p = 0.953), marital status (p = 0.771) or educational level (p = 0.971). Additionally, the International Index of Erectile Function-5 (IIEF-5) score was not different between the two groups, which indicated that the erectile function of the lifelong PE patients was preserved. However, the Chinese Index of Premature Ejaculation (CIPE) scores and intravaginal ejaculatory latency time (IELT) revealed a significant difference between the two groups (NC: 22.07 ± 2.02, PE: 9.00 ± 2.12, p < 0.01 and NC: 10.69 ± 6.84 min, PE: 0.86 ± 0.41 min, p < 0.01, respectively). Furthermore, each question from the CIPE also revealed a significant difference between the lifelong PE patients and NC subjects (Table 1).

**Table 1:** Demographic and clinical characteristics of patients and control participants.

	PE (n = 20)	NC (n = 15)	p
**Age (years)**			
*Mean* ± *SD*	27.95 ± 4.52	27.87 ± 3.78	0.953
**Marital status (%)**			0.771
*Single/Married*	55/45	60/40	
**Education level (%)**			0.971
*Elementary*	20.00	13.33	
*High school*	50.00	40.00	
*University*	30.00	46.67	
**IIEF-5 score**			
*Mean* ± *SD*	23.75 ± 1.25	24.13 ± 0.74	0.300
**CIPE**			
Total	9.00 ± 2.12	22.07 ± 2.02	<0.01
Q1	1.95 ± 0.76	5.00 ± 0.00	<0.01
Q2	1.15 ± 0.37	4.13 ± 0.74	<0.01
Q3	1.25 ± 0.44	4.13 ± 0.74	<0.01
Q4	1.20 ± 0.41	4.13 ± 0.64	<0.01
Q5	3.45 ± 1.54	4.67 ± 0.49	<0.01
**IELT(in min)**			
*Mean* ± *SD*	0.86 ± 0.41	10.69 ± 6.84	<0.01

The data from questionnaires are presented in terms of the mean scores (Mean) and standard deviation (SD) in the premature ejaculation (PE) and healthy control (NC) groups.

IIEF-5 = International Index of Erectile Function-5; CIPE = Chinese Index of Premature Ejaculation; IELT (Q1), sexual satisfaction (Q2), partner’s sexual satisfaction (Q3), frequency of maintaining erection to complete sexual intercourse (Q4), frequency of feeling anxious, depressed or stressed about sexual activity (Q5); IELT = intravaginal ejaculatory latency time.

### Between-group activation differences according to the task fMRI results

**Figure 1:**
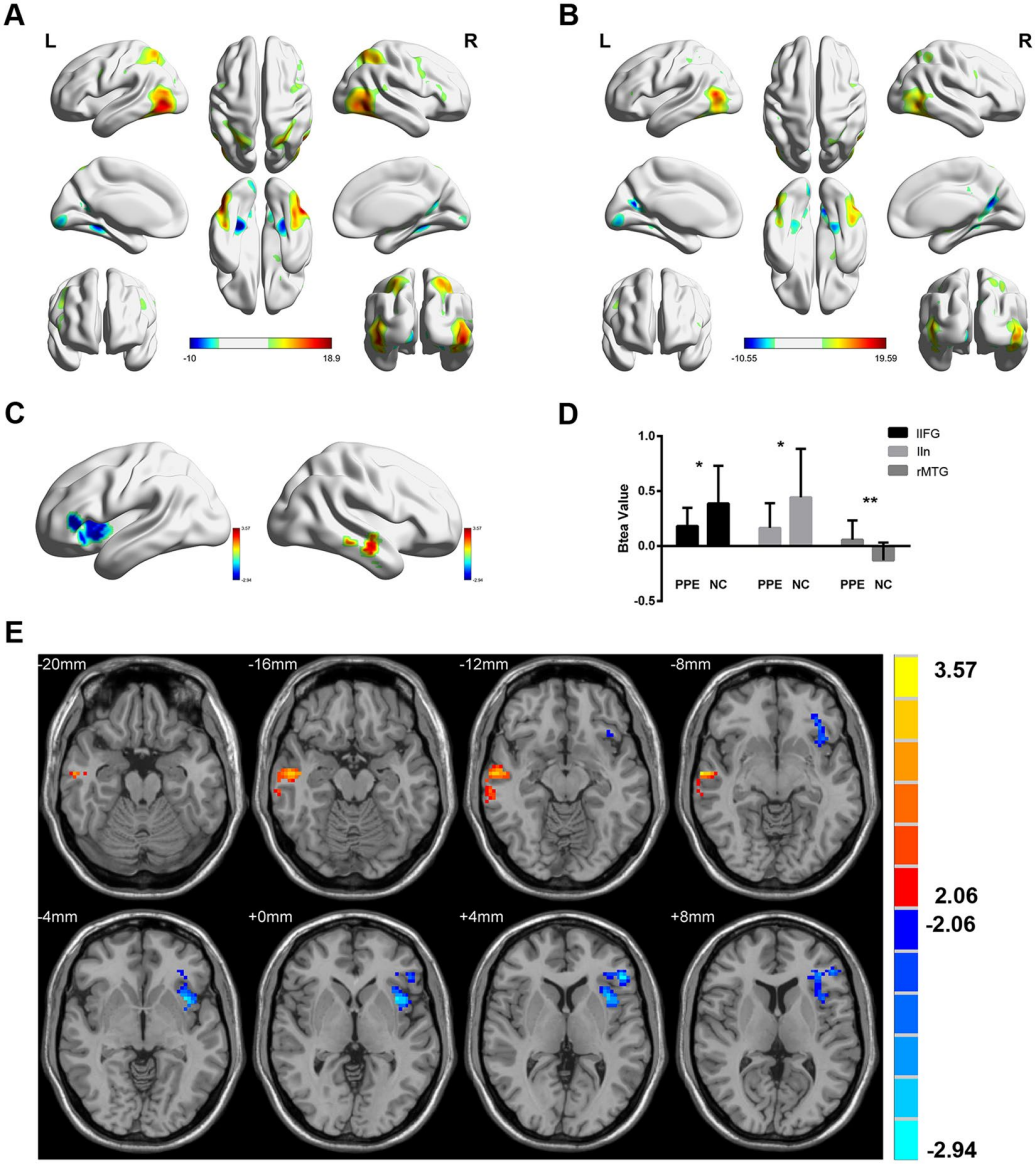
Brain responses to erotic pictures. The mean task fMRI activation maps of (**A**) PE patients and (**B**) NC subjects. (**C,E**) Independent sample t-test results indicating the voxels in which the PE patients demonstrated significantly altered activation compared to the NC group (T maps threshold at p < 0.05, AlphaSim corrected). (**D**) The beta values for the left inferior frontal gyrus, left insula and right middle temporal gyrus. rMTG: right middle temporal gyrus; lIFG: left inferior frontal gyrus; lIn: left insula.

The brain activations to erotic picture stimuli were evaluated using the scenery pictures as a baseline. Both groups demonstrated widespread bilateral activation of the frontal lobe, temporal lobe, parietal lobes, and thalamus (Fig. 1A,B). The between group comparison showed that the brain activation in the left inferior frontal gyrus (lIFG, x, y, z = −50, 34, 6, T = −2.86, p < 0.05, AlphaSim corrected) and left insula (lIn, x, y, z = −36, 12, −3, T = −2.94, p < 0.05, AlphaSim corrected) of the lifelong PE patients significantly decreased and that the signals in the right middle temporal gyrus (rMTG, x, y, z = 60, −18, −18, T = 3.57, p < 0.05, AlphaSim corrected) increased when the patients were stimulated with the erotic pictures (Fig. 1C,E). The beta values in the lIFG, lIn and rMTG are shown in Fig. 1D.

### Between-group ReHo differences from the resting state fMRI

**Figure 2:**
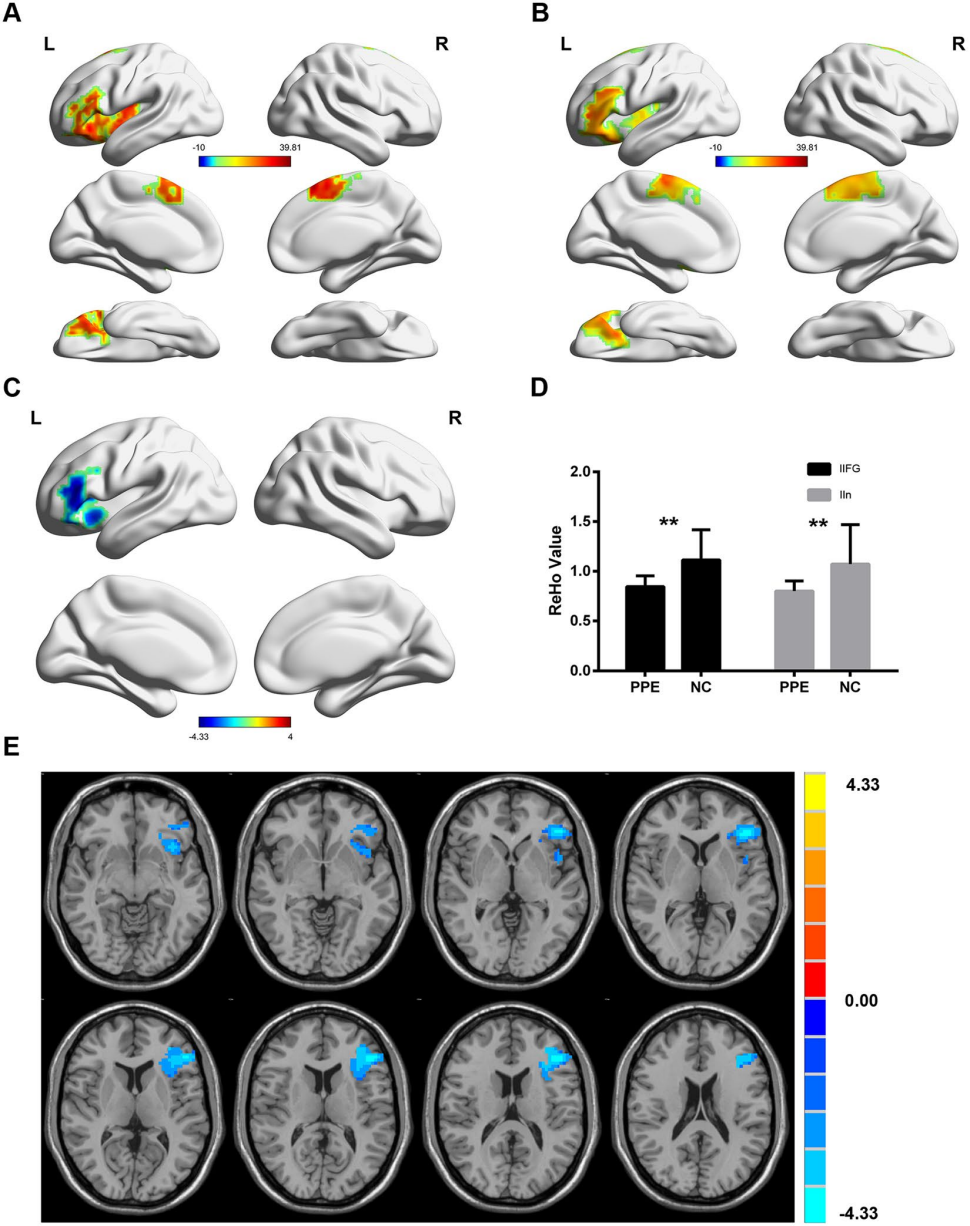
Resting brain spontaneous activity. The mean rs fMRI activation maps (ReHo) of (**A**) PE patients and (**B**) the NC group. (**C,E**) t-test results of independent samples indicating the voxels in which the PE patients demonstrated significantly decreased activation compared to the NC group (T map threshold at p < 0.05, AlphaSim corrected). (**D**) The ReHo value in the left inferior frontal gyrus and left insula. lIFG: left inferior frontal gyrus; lIn: left insula.

In accord with the decreased response found in certain brain areas during the visual picture stimuli, both groups demonstrated widespread bilateral activation of the frontal lobe, temporal lobe, parietal lobes, and thalamus (Fig. 2A,B). For between group comparison, we observed that the lIFG and lIn had lower ReHo value among lifelong PE patients, which indicated that the lifelong PE patients not only had a brain function impairment in the task state but also exhibited a pre-existing change in the spontaneous brain activity pattern at rest. The ReHo values in the lIFG and lIn are shown in Fig. 2C–E. The decreased ReHo in PE patients within lIFG and IIn were in line with the decreased activation to sexual stimuli in the same regions. However, after measuring the ReHo values, we did not find that the rMTG activity was increased in the PE patients in a state of rest.

### Functional connectivity altered in the resting state

**Figure 3:**
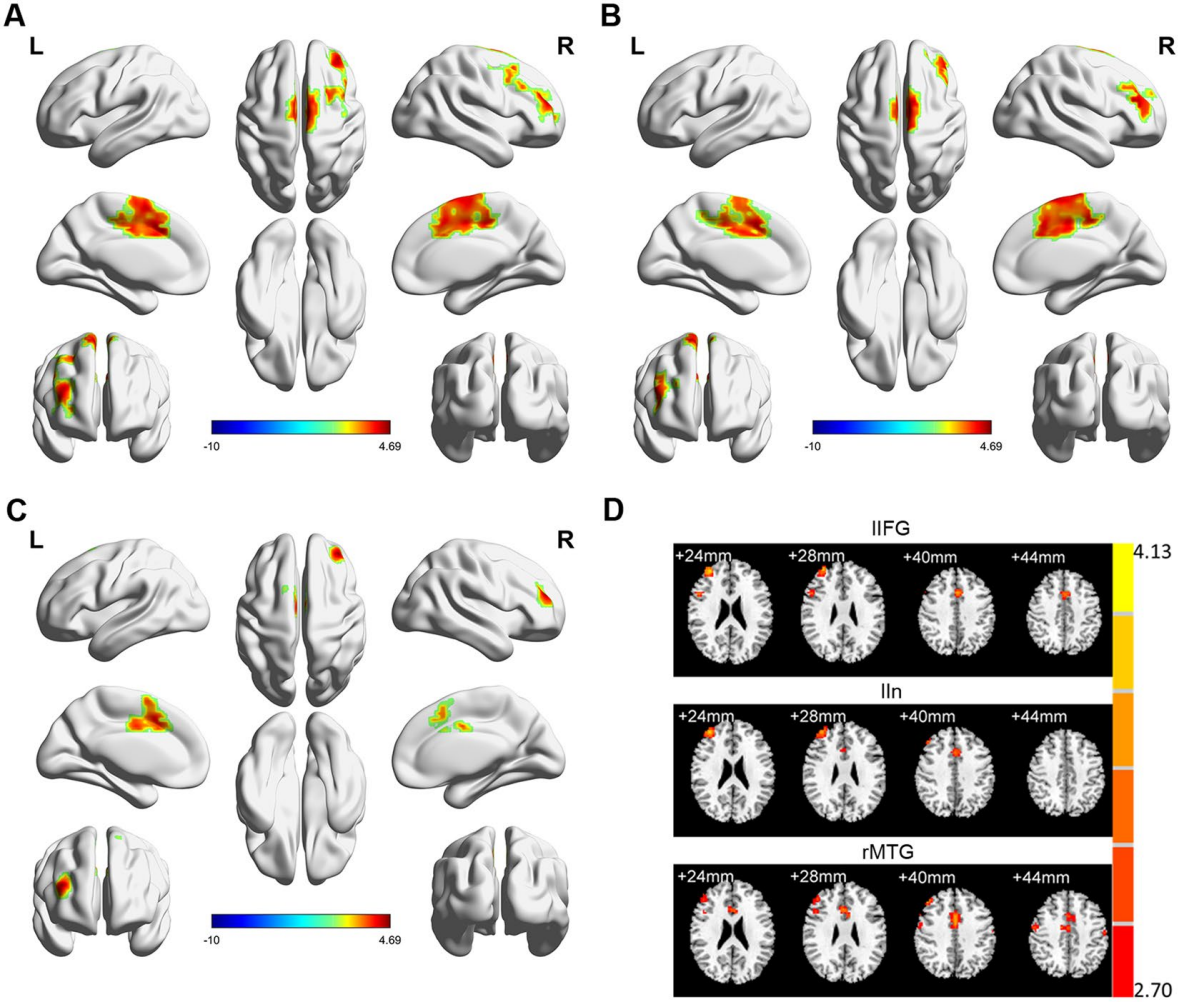
Functional connections altered in the resting state. Based on using the (**A**) lIFG, (**B**) lIn and (**C**) rMTG as seeds, the functional connectivity was enhanced in the PE patients compared with the NC group. An independent sample t-test was applied between the two groups (T maps threshold at p < 0.05, AlphaSim corrected). (**D**) The brain areas where functional connectivity was enhanced in the left inferior frontal gyrus and left insula. rMTG: right middle temporal gyrus; lIFG: left inferior frontal gyrus; lIn: left insula.

We next used each of the brain areas identified above (the rMTG, lIFG and lIn), in which the brain activity was altered both in the task and resting states, as seeds to calculate voxel-wise FC maps of the entire brain using a GLM analysis. The result indicated that the rMTG had a strengthened FC with the middle cingulate cortex (MCC), right middle frontal gyrus (rMFG) and supplementary motor area (SMA) (p < 0.05), while the lIFG and lIn had a significantly enhanced FC with the MCC, rMFG and SMA (p < 0.01) (Table 2, Fig. 3).

**Table 2:** Functional connectivity value of three areas in premature ejaculation patients and healthy controls.

Brain area		FC (MCC)	FC (rMFG)	FC (SMA)
rMTG	PE	0.45 ± 0.21	0.40 ± 0.18	0.50 ± 0.18
	NC	0.33 ± 0.11	0.30 ± 0.14	0.38 ± 0.13
	t	2.089*	1.840*	2.187*
lIFG	PE	0.44 ± 0.19	0.40 ± 0.15	0.53 ± 0.18
	NC	0.27 ± 0.17	0.25 ± 0.20	0.33 ± 0.16
	t	2.836**	2.502**	3.367**
lIn	PE	0.88 ± 0.19	0.45 ± 0.21	0.69 ± 0.23
	NC	0.56 ± 0.27	0.17 ± 0.16	0.36 ± 0.21
	t	4.241**	4.289**	4.407**

The data from each group are presented in terms of the mean score (Mean) and standard deviation (SD) in the PE and NC groups. MCC: middle cingulate cortex; rMFG: right middle frontal gyrus; SMA: supplementary motor area; rMTG: right middle temporal gyrus; lIFG: left inferior frontal gyrus; lIn: left insula; FC = Functional connectivity; *p < 0.05; **p < 0.01.

### Correlation between clinical scale scores and brain activity

**Figure 4:**
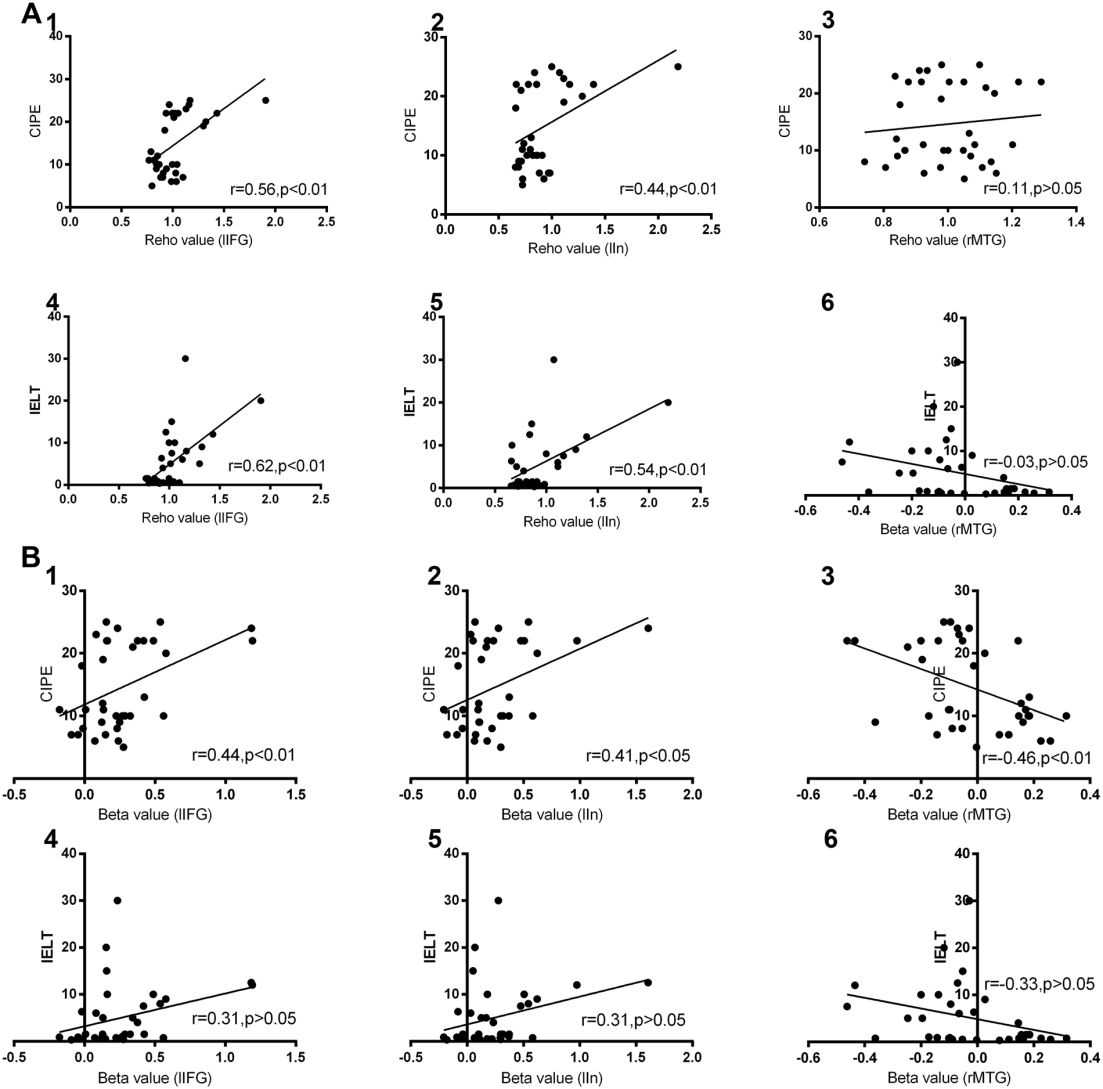
Correlation between clinical scale score and brain activity (ReHo and beta values). (**A**) ReHo, A.1 and A.4: the ReHo value in the lIFG correlated with the CIPE and IELT; A.2 and A.5: the ReHo value in the lIn correlated with the CIPE and IELT; A.3 and A.6: the ReHo value in the rMTG correlated with the CIPE and IELT. (**B**) Beta, B.1 and B.4: the beta value in the lIFG correlated with the CIPE and IELT; B.2 and B.5: the beta value in the lIn correlated with the CIPE and IELT; B.3 and B.6: the beta value in the rMTG correlated with the CIPE and IELT. rMTG: right middle temporal gyrus; lIFG: left inferior frontal gyrus; lIn: left insula, CIPE = Chinese Index of Premature Ejaculation; IELT = intravaginal ejaculatory latency time.

We extracted the activation beta, FC and ReHo values of the rMTG, lIFG and lIn in the lifelong PE patients and NC subjects (Fig. 4A,B). We found statistically significant positive correlations between the Reho value and the scores from the following tests: the IELT test (r_lIFG_ = 0.62, p < 0.01; r_lIn_ = 0.54, p < 0.01; r_rMTG_ = −0.03, p > 0.05) and the CIPE-5 test (r_lIFG_ = 0.56, p < 0.01; r_lIn_ = 0.44, p < 0.01; r_rMTG_ = 0.11, p > 0.05). In addition, there was a significant positive correlation between the beta value and the scores from the following tests: the IELT test (r_lIFG_ = 0.31, p > 0.05; r_lIn_ = 0.31, p > 0.05; r_rMTG_ = −0.33, p > 0.05) and the CIPE-5 test (r_lIFG_ = 0.44, p < 0.01; r_lIn_ = 0.41, p < 0.05; r_rMTG_ = −0.46, p < 0.01). The increased FC at resting state of both the lIFG and lIn with the MCC, rMFG and SMA was negatively correlated with the IELT and CIPE test scores (p < 0.01). However, only a strengthened FC of the rMTG with the MCC, rMFG and SMA had a negative correlation with the CIPE test (Table 3). These findings indicated that the lower the clinical scale scores, the worse the sexual performance, and both the beta and ReHo values in the lIFG and lIn had a significant correlation with the IELT and CIPE scores, whereas only the beta value in the rMTG was correlated with the CIPE scores.

**Table 3:** Pearson’s correlation of functional connectivity between three brain areas and clinical scale scores.

	**IELT**	**CIPE-5**	**Q1**	**Q2**	**Q3**	**Q4**	**Q5**
**lIFG**							
FC(MCC)	−0.44**	−0.47**	−0.43**	−0.41*	−0.52**	−0.39*	−0.37*
FC(rMFG)	−0.38*	−0.42*	−0.38*	−0.40*	−0.50**	−0.37*	−0.21
FC(SMA)	−0.55**	−0.55**	−0.468**	−0.51**	−0.58**	−0.50**	−0.39*
**lIn**							
FC(MCC)	−0.426*	−0.49**	−0.43*	−0.41*	−0.49**	−0.46**	−0.41*
FC(rMFG)	−0.37*	−0.45**	−0.33*	−0.42*	−0.51**	−0.42*	−0.23
FC(SMA)	−0.47**	−0.56**	−0.48**	−0.49**	−0.59**	−0.50**	−0.44**
**rMTG**							
FC(MCC)	−0.27	−0.39*	−0.45**	−0.37*	−0.40*	−0.37*	−0.13
FC(rMFG)	−0.31	−0.34*	−0.34*	−0.31	−0.35*	−0.28	−0.21
FC(SMA)	−0.33	−0.41*	−0.43**	−0.42*	−0.41*	−0.43*	−0.11

MCC: middle cingulate cortex; rMFG: right middle frontal gyrus; SMA: supplementary motor area; rMTG: right middle temporal gyrus; lIFG: left inferior frontal gyrus; lIn: left insula; *p < 0.05; **p < 0.01.

## Discussion

In this study, we examined the brain activation to erotic stimuli using task fMRI with a beta value measurement and measuring spontaneous activity at rest using the ReHo and FC method. Our primary findings include: (1) The task and rs fMRI analyses revealed that the PE group demonstrated higher overall activation in the rMTG than the NC group when stimulated with erotic pictures, and in the lIFG and lIn, we found that brain activity stimulated by the erotic pictures and ReHo were both significantly decreased. (2) Additionally, we found that the rMTG, lIFG and lIn had strengthened functional connections to the bilateral MCC, rMFG and bilateral SMA. (3) Furthermore, both the ReHo and task activation in the lIFG and lIn were positively correlated with the IELT and CIPE scores, but only the beta value in the rMTG had a negative correlation with the CIPE.

Male sexual behavior is a motivated and complex behavior. Ejaculation is the result of the culmination of other sexual behavior components^20^. It is also a multidimensional experience involving psychosexual and physiosexual components as well as inhibitory processes that rely on multiple brain regions^21, 22^. Our study found that the erotic picture stimuli led to an increased response in the rMTG. Previous studies have revealed that the temporal lobe interprets the meaning of visual stimuli and establishes object recognition. The ventral part of the temporal cortices appears to be involved in high-level visual processing of complex stimuli and scenes. Those conclusions are consistent with our results obtained with the erotic picture stimuli. After ejaculation, the activity of the temporal lobe increases^9^, which indicates that the temporal lobe plays a very important role in sexual behaviors. The increased signal in the rTMG in the PE patients may cause increased sensitivity to erotic picture stimuli, resulting in a decline in the ejaculation threshold.

In our cohort, the brain activity of the lIFG and lIn both in response to the erotic picture stimuli and at a resting state was consistently decreased in cases of PE. This was an interesting result that indicated that those cortical functions were in disequilibrium. Studies using visual sexual stimuli (VSS) in healthy men have reported activation of the medial prefrontal cortex and insula^23^. The activation of the medial prefrontal cortex has been related both to general emotional arousal^10, 24^ and to a specific role in mediating the erectile response^25^. In addition, activation of the ventral medial prefrontal cortex has been positively correlated with ratings of pleasure^26^. Activation of the insula by sexual stimuli has been observed in numerous previous studies^27–29^. The function of the insula is related to visceral sensory processing, awareness of an erection^30, 31^, and the perception of penile stimulation^32^. The insula also plays a role in mediating penile erection^25, 33, 34^. Our results revealed that the functions of the left prefrontal cortex and insula were damaged based on the observation of decreased signals with the erotic picture stimuli and in the resting state. Waldinger speculated that lifelong PE is mediated by a more complex interaction of the central nervous, peripheral nervous and endocrine systems^35, 36^. Previous studies have found that PE is associated with a serotonergic deficit, and the current pharmacological remedy is a selective serotonin reuptake-inhibitor (SSRI)^35^. Lifelong PE is not just characterized by diminished serotonergic neurotransmission and a disturbance of 5-HT_1A_ and 5-HT_2C_ receptor function, which disturbs serotonergic modulation of the IELT^35^. Graf^37^ and Abler^15^ reported altered brain activation during erotic visual stimulation and attentional preparation following the administration of serotonergic drugs in healthy subjects, while Metzger *et al*.^38^ reported that baseline resting state FC predicted the individual effects on ejaculation of subsequent SSRI treatment. The dysfunctional brain areas in this study overlap with those of previous studies of brain activity both during erotic stimulation and at rest. We speculate that the serotonergic deficit in these PE patients caused the increased BOLD signal in the rTMG and decreased BOLD signal in the lIFG and lIn.

In this study, we identified three altered brain regions (the rMTG, lIFG, and lIn) according to a higher beta value in the rMTG during stimulation with erotic pictures and according to decreased beta and ReHo values in the lIFG and lIn during the task and resting states. Therefore, we employed those three brain areas as seeds in a FC analysis to calculate the temporal correlation between spatially remote neurophysiological events^34^. We then found increased connectivity of the rMTG, lIFG and lIn with the bilateral MCC, rMFG and bilateral SMA. The increased FC between the impaired areas changes the brain integration and may compensate for other functions. The SMG is part of the somatosensory association cortex, which interprets tactile sensory data and is involved in the perception of space and limb location^39^. Thus, the SMG is part of the mirror neuron system and may contribute to the processes directing sexual desire to behavior^40^. The MCC has the highest connectivity with cognitive- and motor-related areas, and activity in the MCC has been strongly correlated with areas involved in sensorimotor processing^41^. Our results also showed that the FCs of the lIFG and lIn with the bilateral MCC, rMFG and bilateral SMA were stronger than the FC of the rMTG with the bilateral MCC, rMFG and bilateral SMA, which may indicate that the lIFG and lIn are more important for processing sexual behavior. The lIFG is located at the lateral OFC and is thought to play a role in the assessment of the sexual relevance of stimuli^22^. We could surmise that the strengthened connection between these regions may reflect a compensation for other functional connections, such as insufficient activation of the inhibitory ejaculation control center of the CNS^12^, which is preferentially affected during the pathological process of PE.

The IELT is the time it takes for a man to ejaculate during vaginal penetration^42^ and is a screening test that is one criterion for diagnosing lifelong PE^2^. The IELT may be relevant in the perception of sexual performance and actual satisfaction. In our study, we discovered that the IELT score was positively correlated with the ReHo value, which means that the lower the IELT scores obtained by patients, the lower the brain response, and this outcome agreed with the results we found above. However, the IELT did not appear to have a correlation with the beta value. The CIPE, which is a useful approach for evaluating patients with PE^43^, had a positive relationship with the beta value in the lIFG and lIn and a negative relationship with that in the rMTG. This result can also be interpreted to mean that the lower the beta value in the lIFG and lIn, the lower the brain response, which lowers the threshold of the spinal ejaculation center^44^. In contrast, a higher beta value in the rMTG would lead to greater sensitivity to erotic stimuli and a lower IELT score. The FCs of the lIFG and lIn with the MCC, rMFG and SMA had a negative correlation with the IELT and CIPE score, while the rMTG had a negative relationship with only the CIPE. This result indicates that the CIPE is more sensitive to the rMTG than the IELT regarding FC. These findings may indicate a relationship between disease severity and fMRI biomarkers, which could lead to a better understanding of PE using some simple, accessible tools.

The main limitation of this study was that it did not have a longitudinal design. Therefore, these results cannot explain the causality between the symptoms, psychological factors, duration of symptoms and altered cerebral structures. Additionally, the sample size in our study was relatively small. Furthermore, there are several other factors that may be directly associated with male sexual dysfunction, such as fear, personality, and other psychosocial stresses^45^ that should be considered in the task design. We will conduct questionnaires (e.g. MINI or SCID) rather than self-reported psychological disorders in our future study. Last, our fMRI results could pass AlphaSim correction, but could not pass some other strict correction. Maybe it is limited to the small sample. In the future study, we should recruit more participator and use stricter correction method to verify our results.

In conclusion, this study used fMRI to reveal changes in the task activation and functional integration during a task and in a resting state in lifelong PE patients and correlated these alterations with clinical scores for the first time. We believe that these findings enhance the clinical understanding of PE and provide a new approach for future studies and the development of new therapies.

## Materials and Methods

### Subjects

The study was approved by the ethical committee of the Affiliated Drum Tower Hospital of Nanjing University Medical School, and the methods were carried out in accordance with the approved guidelines. From 2012 to 2014, twenty right-handed heterosexual outpatients with lifelong PE patients were included in the study. All participants were recruited from the clinic of Andrology Department in the Affiliated Drum Tower Hospital of Nanjing Universtiy, Nanjing, China.

The lifelong PE patients were diagnosed according to ISSM guidelines^46^: 1) ejaculation that always or nearly always occurs prior to or within about 1 minute of vaginal penetration; b) the inability to delay ejaculation on all or nearly all vaginal penetrations; c) negative personal consequences such as distress, bother, frustration, and/or the avoidance of sexual intimacy.

For comparison, fifteen right-handed healthy subjects were enrolled in this study as controls, with self-reported IELTs of more than three min. The IELT was measured for the 4-week baseline period during which both patients and normal potent men were asked to have sexual intercourse at least 4 times. All the subjects were in a stable relationship with the same, non-pregnant, sexually active partner for at least 1 year.

We excluded subjects with erectile dysfunction, a genitourinary tract infection, systemic or neurological problems or self-diagnosed psychological disorders. Prior to participation in the study, all the eligible participants received a comprehensive andrological diagnostic workup, which included obtaining a detailed medical history, physical examination, and hormonal evaluation to rule out another potential source of reported sexual problems. Furthermore, we excluded subjects with the presence of structural abnormalities that could cause cognitive impairment, which were identified by conventional MRI.

All the subjects completed the following two questionnaires: the CIPE, with five questions^43^, and the IIEF-5^47^. We explained the study design in detail to all the included subjects, and they signed an informed consent form.

## Data Acquisition

### Stimuli and the experimental design paradigm

**Figure 5:**
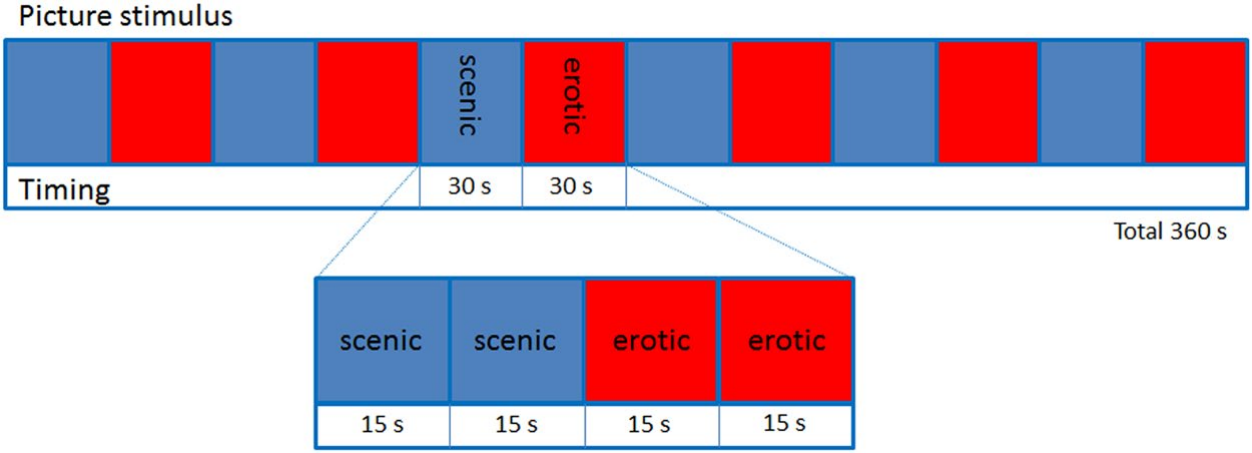
Erotic picture stimuli task. The blue bars represent the scenic pictures, and the red bars represent the erotic pictures. Subjects first saw two random scenic pictures for 15 s each (blue pictogram). Then, the participants saw two random erotic pictures, also for 15 s each (red pictogram). These two sections were counted as one set, and there were six sets in the whole paradigm for a total of 360 s.

The task stimulus was presented to the subjects via a visual stimulation system (VSS) for fMRI (Sinorad Medical Electronics Co., Ltd., Type SA-9900, Shenzhen, China), and the E-prime software (Psychology Software Tools, Inc.) was used to collect the behavioral data. The erotic pictures were presented in 30-s blocks separated by 30-s intervals of scenic pictures. Each erotic block and scenic block contained two pictures, and the pictures were alternately presented six times. A total of 12 erotic and 12 scenic pictures were included in this paradigm (Fig. 5). All the pictures were selected from the International Affective Picture System (IAPS)^48^. The valence of the erotic pictures in IAPS had no significant difference between young male participants from China and America (**χ** ^**2**^ = 1.149, p = 0.700)^49^. The erotic pictures from IAPS were chosen with the intention of inducing maximum appetence in the viewer and included either pictures of single naked subjects or pictures of couples in an intimate situation. The tasks and instructions were presented on a back projection screen and were viewed through a mirror mounted onto the 8-channel head coil. Prior to completing the task in the scanner, a practice session was given to ensure task familiarity and capacity (i.e., to ensure that the participants did not have significant visual or motor impairment that precluded performance).

### MRI protocol

The fMRI experiment was performed with a 3 T MR system (Achieva 3.0 T TX, Philips Medical Systems, Eindhoven, Netherlands) using an 8-channel phased array coil. The subjects were positioned in the supine position in a dark environment with their heads elevated 10–15° from the horizontal level. Their heads were fit into a head restrainer to minimize motion and to provide precise positioning and comfort. The fMRI scan was acquired from the entire brain using an echo planar imaging (EPI) method with a SENSE factor = 2, TR/TE/FA = 2000 ms/30 ms/90°, FOV = 192 mm × 192 mm × 140 mm, slice thickness = 4 mm with no distance between slices, repetition = 180, and acquisition time = 6 min. A resting state fMRI was acquired for the entire brain using an echo planar imaging (EPI) method with a SENSE factor = 2, TR/TE/FA = 2000 ms/30 ms/90°, FOV = 192 mm × 192 mm × 140 mm, slice thickness = 4 mm with no distance between slices, repetition = 230, and acquisition time = 7 min 40 s. A high-resolution T1-weighted structural scan was also acquired for each participant, TR/TE/FA = 7600 ms/3400 ms/8°, FOV = 256 mm × 256 mm × 178 mm, slice thickness = 0.8 mm with no distance between slices, and acquisition time = 7 min 18 s.

## Data Analysis

### fMRI Data Processing

The fMRI data were processed with SPM8 software (Wellcome Department of Imaging Neuroscience, University London College, UK^50^). The first ten images of each fMRI data set were discarded to remove the initial transit signal fluctuations. The subsequent images were realigned within the session to remove any minor head movements. The T1-weighted high-resolution anatomical images were co-registered and spatially normalized to the Montreal Neurological Institute (MNI) brain template^51^ with a spatial resolution of 1 mm × 1 mm × 1 mm. The time-course images were spatially normalized using the same normalization parameters with a spatial resolution of 3 mm × 3 mm × 3 mm and then smoothed with an 8 × 8 × 8 mm^3^ (full width at half maximum, FWHM) Gaussian smoothing kernel.

A statistical parametric map was generated for each subject under the erotic picture stimulus condition by fitting the stimulation paradigm to the functional data, which were convolved with an HRF and time derivative. The voxels representing the active structures were overlaid on the 3D T1-weighted anatomical image with MNI coordinates. A group analysis was conducted to generate an average activation map for each study group.

### Hemodynamic response function

An HRF was applied to analyze the BOLD task fMRI data in the SPM8 software (Wellcome Department of Imaging Neuroscience, University London College, UK^50^) with a standard analysis pipeline. The hemodynamic response (HR) allows the rapid delivery of blood to active neuronal tissues. A beta value represented the HRF.

### ReHo measurement

ReHo methods allow for the impartial examination of spontaneous neural synchronization at rest^18^. ReHo can provide a rapid method for mapping regional activity across the entire brain^18^. In the current study, individual ReHo maps were generated by calculating Kendall’s coefficient of concordance (KCC) between the time series of each given voxel and those of its nearest neighbors in a voxel-wise manner. For a given voxel,

$${\rm{ReHo}}=\frac{{\sum }^{}{({R}_{i})}^{2}-n{(\bar{R})}^{2}}{{k}^{2}({n}^{3}-n)/12}$$

where the ReHo is calculated using the KCC. The ReHo ranges from 0 to 1, and the higher the ReHo, the higher the similarity of the local activity of a given voxel is to that of its neighbors. ${R}_{i}=\sum _{j=1}^{k}{r}_{ij}$ is the sum rank of the *i*−*th* time point, and *r*_*ij*_ is the rank of the *i*−*th* time point of the *j*−*th* voxel; $\bar{R}$ is the mean of the *R*_*i*_; n is the length of the time series; and *k* is the number of voxels (k = 27 in the present study) within the measured cluster. To reduce the effect of individual variability, we normalized the ReHo value of each voxel by dividing it by the mean ReHo of the entire brain for each subject. For each voxel, ReHo_normlized_ = ReHo_(x, y, z)_/Mean(ReHo). The ReHo calculation was performed using RSET (http://restfmri.net/forum/index.php)^52^.

### Seed-based functional connectivity analyses

FC was computed using a conventional seed-based correlation analysis method. This method involved (1) choosing a specific voxel or cluster of voxels in the brain, which was referred to as the seed, (2) extracting the time series of the BOLD signal in this seed region, and (3) using this time series as a regressor in a GLM analysis to calculate voxel-wise FC maps of the entire brain^53^. Our seed regions of interest were chosen as those clusters that were significantly activated during the erotic picture stimuli task BOLD in the PE patients compared to an NC group. REST (http://restfmri.net/forum/index.php) ^52^ was used in FC calculation.

### Relationship between the ReHo, beta, and FC values and the clinical variables

To determine whether the ReHo index and beta value varied with disease progression in the NC subjects and the PE patients, correlation analyses between the fitted ReHo index, the beta value and each of the clinical variables (CIPE and IELT) were performed. Because these analyses were exploratory in nature, we used a statistical significance level of p < 0.05.

### Statistical Analysis

Statistical analysis was performed using the SPSS software version 16.0 (statistical program for social sciences, SPSS Inc. Chicago, IL) for the demographic and clinical data, and SPM8 (statistical parametric mapping, http://www.fil.ion.ucl.ac.uk/spm) was used for the fMRI data. Two-sample t-tests were used to compare the brain activation between the PE and NC groups (minimum statistical threshold p < 0.05, AlphaSim corrected). A region of interest analysis was conducted on the BOLD responses for each condition in relation to the sex-related scores. Analyses were completed for the entire cohort and separately for the PE and NC groups.
